# Commonly used adjuvants (liquid soap, foam sanitizer, or ultrasound gel) do not improve strength or curing time of fiberglass cast material

**DOI:** 10.1186/s13018-019-1202-1

**Published:** 2019-05-30

**Authors:** Matthew R. I. Meng, Joseph W. Elphingstone, Margaret A. Sinkler, Bruce M. Byrd, Meghan E. McGee-Lawrence

**Affiliations:** 1 Department of Orthopaedic Surgery, Medical College of Georgia, Augusta University, Augusta, GA 30912 USA; 2 Department of Cellular Biology and Anatomy, Medical College of Georgia, Augusta University, 1120 15th St, Augusta, GA 30912 USA

**Keywords:** Fiberglass, Cast, Orthopedics, Fracture, Mechanics, Strength

## Abstract

**Background:**

Bone fractures are one of the most common injuries in the USA. Fiberglass tape is a commonly used casting material, and many medical professionals apply adjuvants including liquid hand soap, foam sanitizers, and ultrasound gel in the hopes of improving outcomes relating to ease of molding and eventual strength, lamination, and smoothness of cast material. However, the efficacy of these agents to improve fiberglass cast mechanics has not been scientifically evaluated. The purpose of this study was to assess the mechanical effects of commonly used adjuvants on fiberglass cast materials.

**Methods:**

Studies compared regularly shaped samples of water-activated, untreated fiberglass tape (Ossur Techform Premium) to water-activated fiberglass tape treated with one of three commonly used adjuvants (liquid soap, foam hand sanitizer, or ultrasound gel) during lamination. Material stiffness, yield stress, and ultimate load were measured by 3-point bending.

**Results:**

These studies demonstrated that that liquid soap and ultrasound gel did not affect fiberglass tape mechanical properties, but alcohol-based foam sanitizer significantly reduced stiffness (− 32.8%), yield stress (− 33.6%), and ultimate load (− 31.0%) of the cast material as compared to the control group. Regression slopes were not significantly different between groups, suggesting that no adjuvants improved material curing time.

**Conclusions:**

These data suggest that the application of adjuvants is not beneficial and potentially harmful to fiberglass cast behavior. Despite the widespread practice of adjuvant application by medical professionals during casting, results from the current study suggest that use of these agents for structural enhancement of fiberglass casts is not beneficial and should largely be discouraged.

**Electronic supplementary material:**

The online version of this article (10.1186/s13018-019-1202-1) contains supplementary material, which is available to authorized users.

## Introduction

Bone fractures are among the most prevalent injuries treated in orthopedic, emergency department, and family medicine clinics, accounting for roughly 14 million injuries annually in the USA [1]. Depending on the severity, classification, and location of a fracture, the preferred treatment modality ranges from a simple splint and bandage to surgical intervention with plates and screws. The majority of closed fractures, with the help of closed reduction, can be immobilized with a cast. Casts and splints have been used for stabilizing fractures for millennia, though the advent of the modern cast using plaster of Paris (POP) occurred sometime after the turn of the eighteenth or early nineteenth century [2]. While POP is still a mainstay in Emergency Departments, fiberglass tape has emerged as the preferred standard of care for many physicians. The popularity of fiberglass in clinics is attributed to its improved strength to weight ratio, decreased exothermic activity, reduced drying times, increased radiolucency, and water resistance [3–7]. These characteristics make fiberglass lighter and stronger and reduce the risk for skin burns. Additionally, patients often request fiberglass casts because they are more comfortable and cause less interference with daily activities [8].

Although not explicitly recommended by manufacturers, many healthcare professionals report applying solutions such as liquid hand soap, foam sanitizer, and water-based gels to fiberglass casts as they set in an effort to improve factors including ease of molding, curing time, cast smoothness, and stiffness [8, 9] (Additional file 1: Table S1). To our knowledge, there have been no studies to date testing the validity or efficacy of these adjuvants for improving cast mechanical behavior. Therefore, the goal of the current study was to address this gap by determining the effect of commonly used adjuvant agents on fiberglass cast material strength and stiffness. Since these adjuvants are not explicitly recommended by manufacturers, we hypothesized that commonly applied agents, such as liquid hand soap, alcohol-based foam sanitizers, or ultrasound gel would provide no significant improvement in fiberglass cast strength and setting time. To assess this, we compared the stiffness, yield stress, and ultimate load of activated fiberglass tape 5, 10, and 15 min after the initial application of adjuvants to the untreated control. We demonstrated that liquid soap and ultrasound gel did not significantly alter cast mechanics along any of the time points or force measures relative to the control. Foam hand sanitizer, however, significantly weakened each force measurement at every time point. The results demonstrate the limited-to-negative utility of these adjuvants on cast mechanics and would thus be discouraged for clinical use for this purpose.

## Materials and methods

All materials selected for study were chosen due to their routine use in the Augusta University Medical Center.

### Fiberglass tape sample preparation

**Fig. 1:**
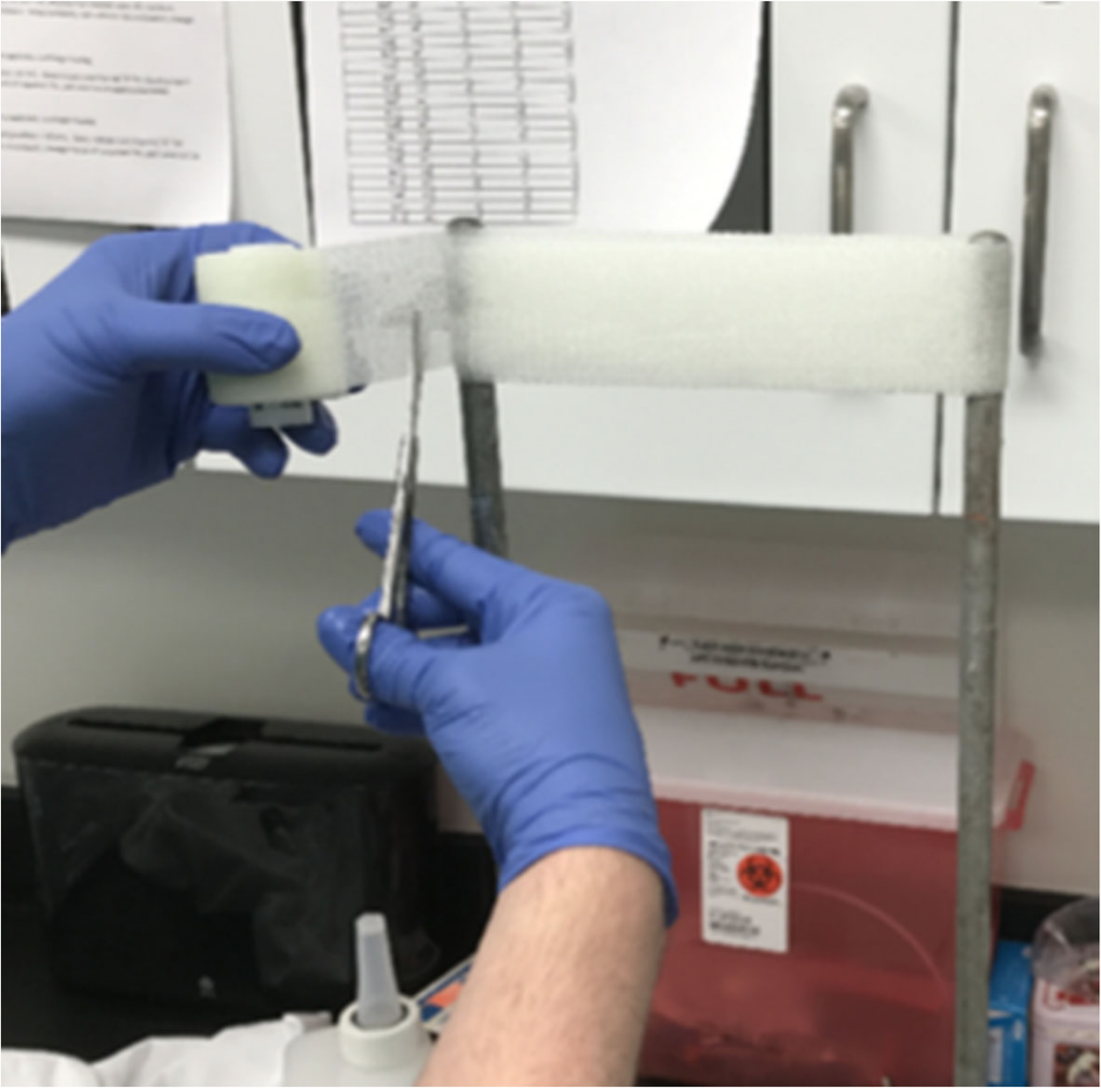
Preparation of fiberglass cast tape samples for testing. After a brief immersion in water, each roll of fiberglass cast material was wrapped around a custom jig (200 mm width) three times to generate a 6-layered sample

Each roll of fiberglass tape (Techform Premium, 2 in. width, Ossur Americas, Orange County, CA, USA) was immersed in 20 °C water for 10 s, as indicated by the manufacturer’s instructions. Water was changed between each roll to prevent potential artifact from resin carry over [10]. Once removed from the water, rolls were briefly allowed to drain excess fluid. Fiberglass tape samples for study were created by wrapping the roll around a 200-mm-wide jig three times (Fig. 1), creating a 6-layered slab of fiberglass tape (average sample depth 4.0 ± 0.4 mm). The cast material was then removed from the stand and compressed slightly to initiate lamination. Three samples were created in this fashion from each roll, and 60 rolls of fiberglass tape were used, generating 180 samples in total for study. Samples were randomized into one of four treatment groups and one of three time points (*n* = 15 per treatment group and time point) so that each adjuvant at each time point was tested from all portions of the tape roll (beginning, middle, and end).

### Adjuvant application

Adjuvants selected for study included ultrasound gel (Aquasonic 100 Ultrasound Transmission Gel, Parker Laboratories, Fairfield, NJ USA), foam hand sanitizer (Soft ‘N Sure Hand Sanitizer, 1381-36, Steris Corporation, Mentor, OH USA), and liquid soap (Acute-Kare Healthcare Personnel Handwash, 1206-87, Steris Corporation, Mentor, OH USA). Three grams of each adjuvant was applied to one side of the sample using gloved hands and a side-to-side motion with consistent pressure for 1 min, after which the sample was turned over and the process repeated on the other side. Control samples were treated in a similar fashion with no adjuvant applied. After lamination, samples were randomly assigned to rest for 5, 10, or 15 min prior to testing.

### Mechanical testing

**Fig. 2:**
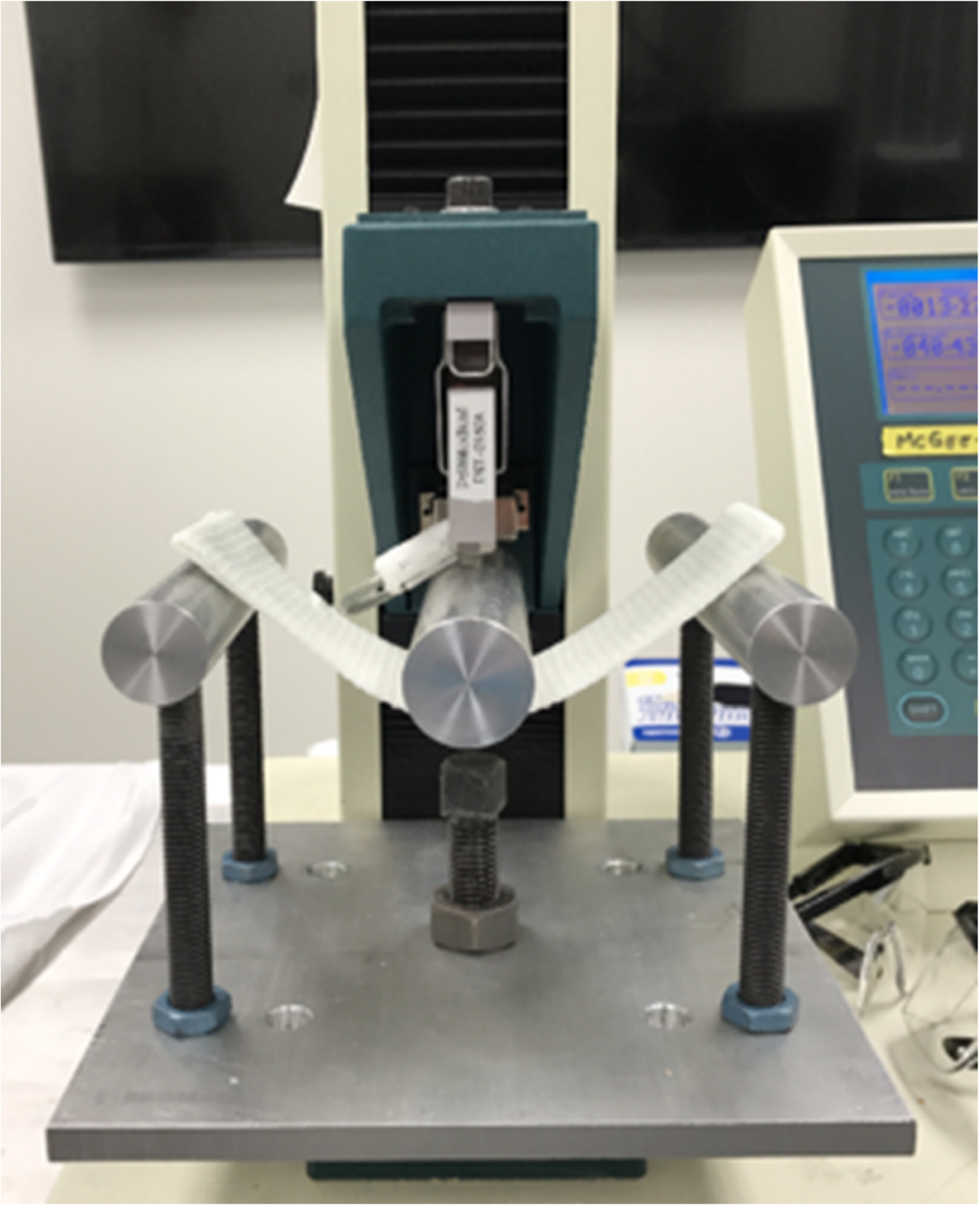
Mechanical testing setup. After adjuvant application and lamination, each sample was centered on a custom 3-point bending fixture (span = 150 mm) and loaded in displacement control at a rate of 100 mm/min to a final displacement of 40 mm

Sample stiffness, yield stress, and ultimate load were assessed via 3-point bending 5, 10, and 15 min after initial adjuvant application with a H5KS mechanical testing system (Tinius Olsen, Horsham, PA, USA) featuring a 500-N force transducer (Fig. 2). The lower span between supports was 150 mm, and experiments were run in displacement control at a rate of 100 mm per minute. Since pilot testing revealed that catastrophic failure (fracture) would not occur under these experimental conditions, experiments were terminated upon reaching a displacement of 40 mm. Load and displacement data were recorded using Horizon software (Tinius Olsen). Ultimate load was defined as the maximum force sustained during testing. Stiffness was calculated as the slope of a trend line fit to 10 randomly selected data points within the linear region of the load-displacement curve. Yield stress was calculated using beam bending theory, an average sample depth of 4 mm, and the 0.2% offset method, as we have previously described [11–13].

### Statistical analysis

Groups were compared by analysis of covariance (ANCOVA) and Tukey’s HSD post hoc testing using JMP Pro 13.0.0 software (SAS, Cary, NC, USA), where time was treated as a continuous variable. Statistical significance was set at *p* < 0.05 for all comparisons. Data are presented on graphs showing each mechanical property vs. time point (5, 10, or 15 min), and each data point shown represents an independent sample. To better highlights trends in the data, a linear regression line of best fit was created for each group and is shown with its 95% confidence interval (shaded). Data points, best-fit lines, and confidence intervals are presented in the same color for each group as follows: blue = control, red = foam sanitizer, green = liquid soap, and purpose = ultrasound gel.

## Results

### Fiberglass cast biomechanical properties linearly increase with curing time

**Fig. 3:**
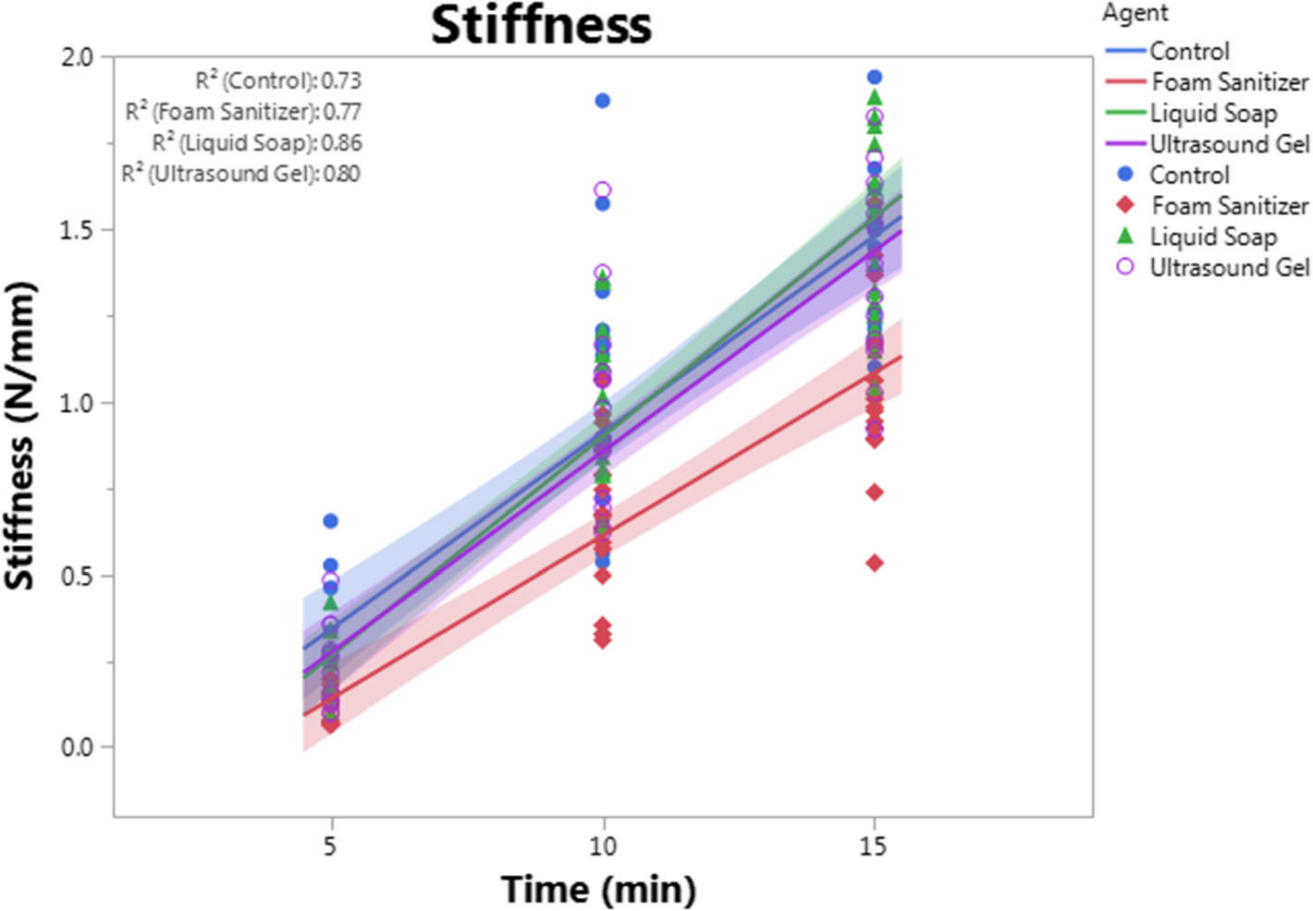
Stiffness. Stiffness was calculated as the slope of the linear portion of the load-displacement curve for each adjuvant group and time point. Samples treated with foam sanitizer had significantly (*p* < 0.0001) reduced stiffness as compared to control, liquid soap, and ultrasound gel groups at each time point, but other groups were not different from one another. Each data point represents an independent sample. A best-fit line with confidence interval (represented by the shaded area) is plotted for each group, with the *R*^2^ values for each line displayed in the upper left of the graph. Data points, best-fit lines, and confidence intervals are presented in the same color for each group as follows: blue = control, red = foam sanitizer, green = liquid soap, purple = ultrasound gel

**Fig. 4:**
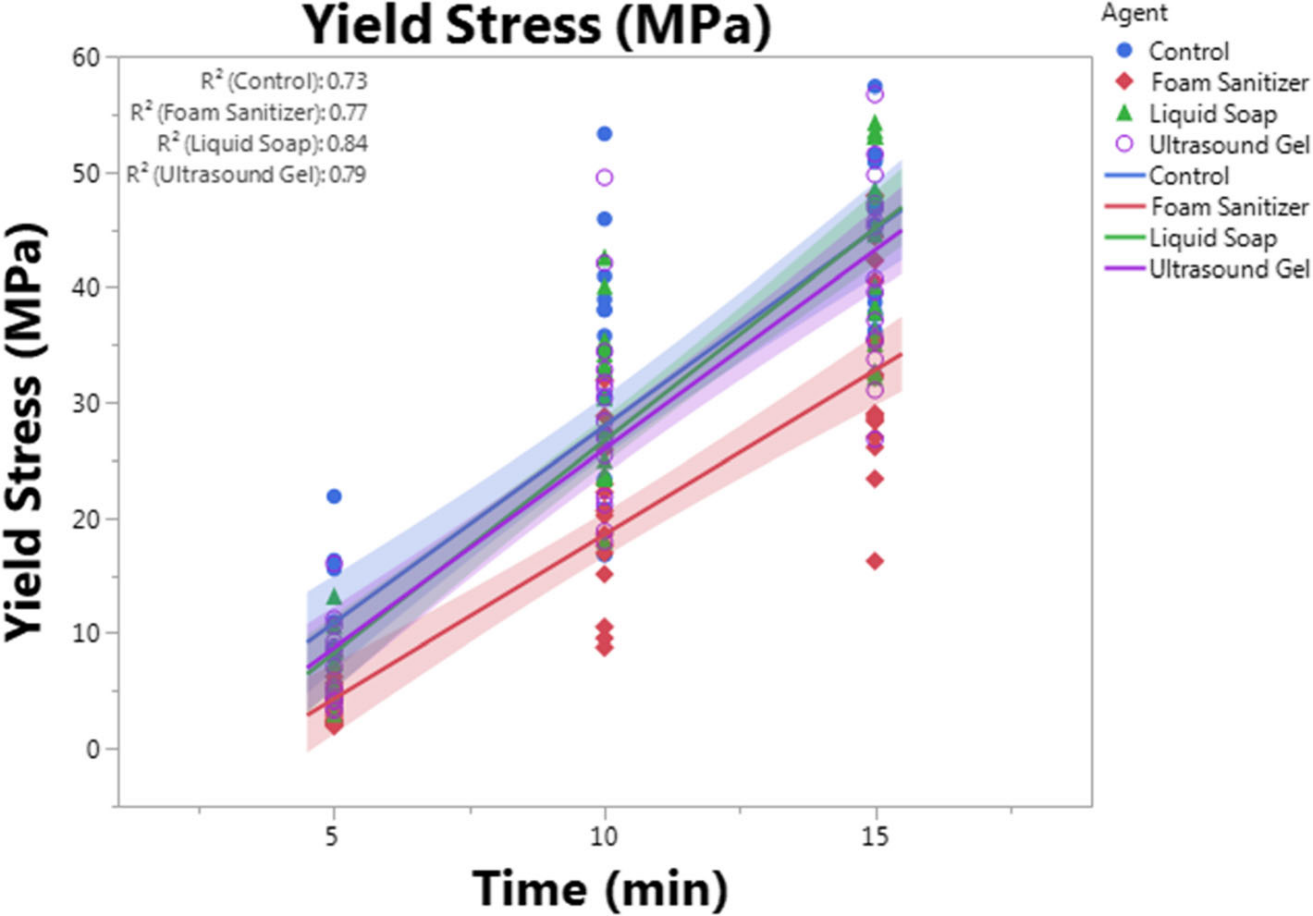
Yield stress. Yield stress was calculated using the 0.2% offset method and an average sample depth of 4 mm for each adjuvant group and time point. Samples treated with foam sanitizer had significantly (*p* < 0.0001) reduced yield stress as compared to control, liquid soap, and ultrasound gel groups at each time point, but other groups were not different from one another. Each data point represents an independent sample. A best-fit line with confidence interval (represented by the shaded area) is plotted for each group, with the *R*^2^ values for each line displayed in the upper left of the graph. Data points, best-fit lines, and confidence intervals are presented in the same color for each group as follows: blue = control, red = foam sanitizer, green = liquid soap, purple = ultrasound gel

**Fig. 5:**
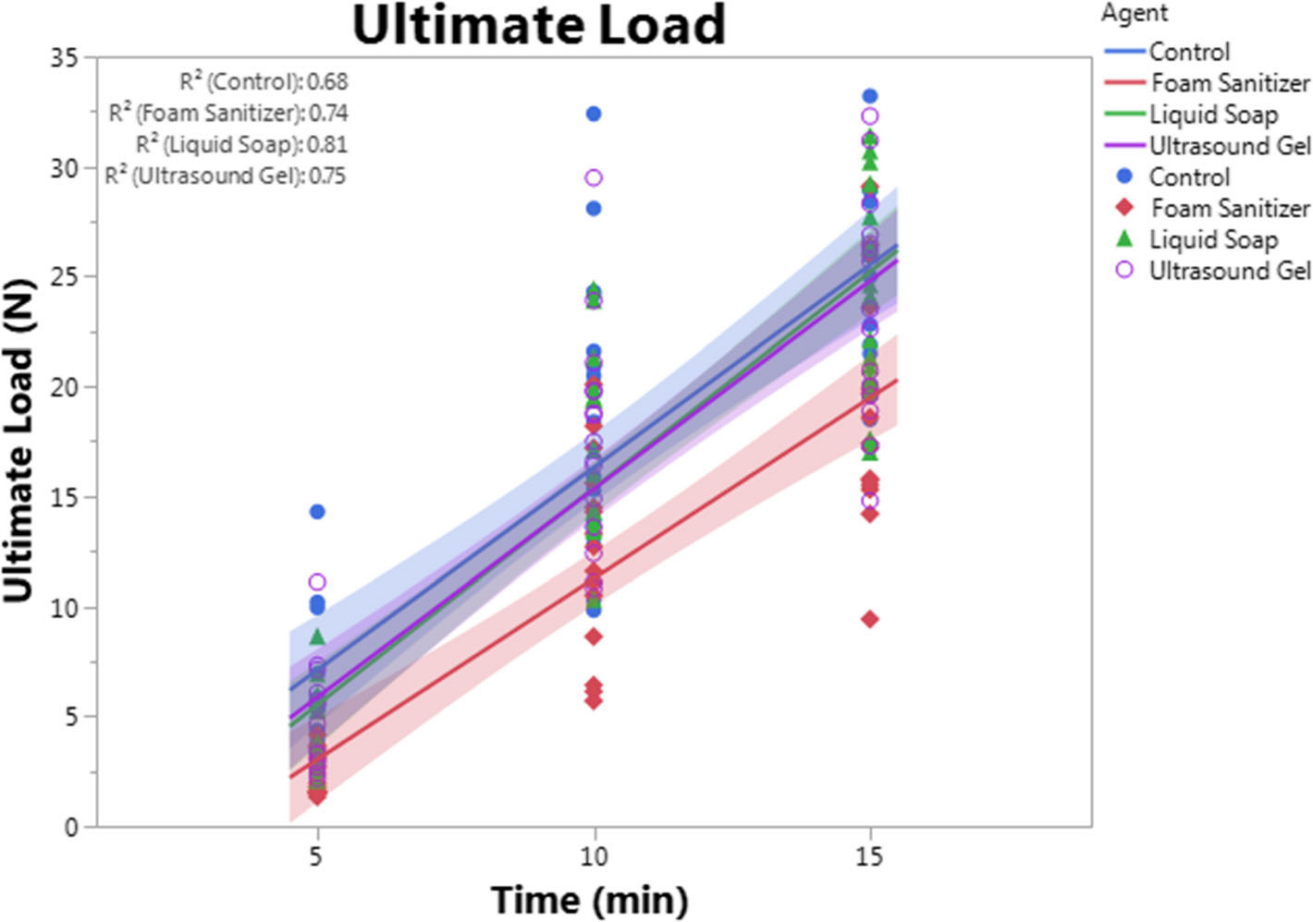
Ultimate load. Ultimate load was calculated as the maximum force sustained during testing for each adjuvant group and time point. Samples treated with foam sanitizer had significantly (*p* ≤ 0.0002) reduced stiffness as compared to control, liquid soap, and ultrasound gel groups at each time point, but other groups were not different from one another. Each data point represents an independent sample. A best-fit line with confidence interval (represented by the shaded area) is plotted for each group, with the *R*^2^ values for each line displayed in the upper left of the graph. Data points, best-fit lines, and confidence intervals are presented in the same color for each group as follows: blue = control, red = foam sanitizer, green = liquid soap, purple = ultrasound gel

To assess the effects of adjuvant application on fiberglass tape mechanical properties, the stiffness, yield stress, and ultimate load for each sample were quantified at 5, 10, and 15 min after lamination. Regardless of treatment group, all biomechanical properties linearly and significantly (*p* < 0.0001) increased over time. For each adjuvant group, the rate of change in each property over time was not different from the control group, suggesting that curing rate was not substantially affected by any adjuvant application (Figs. 3, 4, and 5). Regression analyses indicated that material stiffness increased by an average of 0.11 N/mm/min in the control group. In comparison, stiffness increased by an average of 0.09 N/mm/min in the foam sanitizer-treated samples, by 0.13 N/mm per minute in the liquid soap-treated samples, and by 0.12 N/mm per minute in the ultrasound gel-treated samples. Rates of change for yield stress were 3.41 MPa/min for control, 2.85 MPa/min for foam sanitizer, 3.68 MPa/min for liquid soap, and 3.45 MPa/min for ultrasound gel-treated samples. Rates of change for ultimate load were 1.84 N/min for control, 1.65 N/min for foam sanitizer, 1.97 N/min for liquid soap, and 1.89 N/min for ultrasound gel-treated samples.

### Foam sanitizer impairs fiberglass tape mechanical properties, but liquid soap and ultrasound gel have no effect

Despite their common use during cast application [8, 9], neither liquid soap nor ultrasound gel significantly altered fiberglass tape stiffness (*p* > 0.704), yield stress (*p* > 0.582), or ultimate load (*p* > 0.731) as compared to untreated control samples (Figs. 3, 4, and 5). Interestingly, however, foam sanitizer significantly reduced all quantified properties including stiffness (*p* < 0.0001, − 32.8%), yield stress (*p* < 0.0001, − 33.6%), and ultimate load (*p* < 0.0001, − 31.0%) as compared to control samples. The mechanical properties of the sanitizer-treated samples were also comparably and significantly reduced as compared to soap- or gel-treated samples (*p* ≤ 0.0002) (Figs. 3, 4, and 5).

## Discussion

Anecdotally, the application of adjuvant agents to orthopedic cast material is a common practice by medical professionals [8, 9]. To better assess the current prevalence of adjuvant use during fiberglass cast application, we surveyed 47 orthopedic residents, cast technologists, and attending physicians from the Medical College of Georgia, Atlanta Medical Center, and the University of Washington on their experience with cast application and use of adjuvant agents (the survey questions and responses can be found in Additional file 1: Table S1). Of the polled subjects, 74% (35) stated that they regularly apply fiberglass casts, and of those individuals, 60% (21) regularly employed at least one of the three adjuvant agents studied here. Of those medical professionals who regularly applied adjuvants, 76% (16/21) reported using foam hand sanitizer, 42% (9/21) liquid hand soap, and 23% (5/21) water-based gels like ultrasound gel. The goal of the current study was to assess the impact of adjuvant application on fiberglass cast mechanical properties. We hypothesized, based on clinical observation, that the use of adjuvants like liquid hand soap, foam hand sanitizers, and ultrasound gel would not improve fiberglass cast mechanical properties. Indeed, if adjuvant application would potentially improve cast material behavior, it would be reasonable to expect that the material manufacturer would recommend such treatment during cast application. Results from the current study demonstrate that liquid hand soap and ultrasound gel provided no noticeable benefit for fiberglass cast material stiffness, yield stress, or ultimate load over the time points investigated.

Somewhat surprisingly, foam hand sanitizer did not improve and instead significantly reduced all measured mechanical properties at each time point relative to the other adjuvants and to control-treated samples. The foam sanitizer studied here is largely alcohol-based in composition (62% ethanol). To the best of our knowledge, there have been no studies or reports on the effects of ethanol on fiberglass cast mechanics, but several dental research groups have provided evidence that ethanol pretreatment can weaken fiberglass post bond strength in root implants [14, 15]. This effect was suggested to be the result of weakened epoxy resin, not fiberglass, since silicon dioxide is largely insoluble. The Ossur brand of fiberglass tape used in the current study, like many other brands, is “impregnated” with a water-activated polyurethane resin, which may be alcohol-soluble. It is possible that the weakened mechanical properties observed in sanitizer-treated fiberglass tape samples could be due to cleavage of polyurethane polymers at urethane linkages. This would be consistent with the observation that polyurethane catheters will degenerate and demonstrate impaired mechanical properties following ethanol immersion [16–18]. More work on the molecular interaction between polyurethane resins and ethanol is needed to support this proposed mechanism. Given our results, it would be reasonable to hypothesize that the use of alcohol-based foam sanitizers during fiberglass cast lamination could be linked to greater rates of clinical cast failure, but we are not aware of any such conclusive report at this time.

We demonstrated the negative effect of foam sanitizer on fiberglass tape mechanical properties at 5, 10, and 15 min after adjuvant application. The clinical significance of this has yet to be determined and further testing will be required to reveal whether the mechanical properties remain diminished by the foam sanitizer after 30 min, which is when the cast is described as “fully cured” by the manufacturer, or if these properties may continue to improve over time to approach the mechanical strength of control-treated samples. If the latter is true, foam sanitizer could potentially prove to be a useful tool to delay the achievement of “full mechanical strength” in fiberglass casts that require more time for molding, such as in clubfoot casting. However, if sanitizer-treated cast material failed to eventually achieve a strength comparable to control-treated samples, it would seem wise to discourage the use of alcohol-based sanitizers as an adjuvant during fiberglass cast application.

While many physicians are routinely using adjuvants during the application of fiberglass casts [8, 9] (Additional file 1: Table S1), the results of the current study suggest that these adjuvants have either no impact or a negative effect on the mechanical properties of the cast material. Furthermore, none of the tested adjuvants improved cast material curing time. This raises the question of what economic impact that the unnecessary use of adjuvants may have in the orthopedic field. It would be of interest to conclusively determine the nationwide frequency of adjuvant application during casting and the ultimate effects of such practices on fracture care outcomes. Further studies could provide better insight into the direct and indirect costs of adjuvants, particularly with respect to alcohol-based foam sanitizers.

Our study did have limitations that should be noted. For example, we chose to test regularly shaped slabs of fiberglass cast material rather than anatomic cast molds that would have been more representative of the behavior of casts applied in clinical practice. This choice was made to ensure maximum repeatability across different trials and to facilitate easy calculation of material-based properties like yield stress. Sample depth (6 layers of fiberglass tape) was selected to represent a typical number of lamination layers during clinical casting, but it is important to recognize that the distribution of forces and flexibility of cast material in the slabs studied here is likely not as representative of potential clinical outcomes as an anatomic cast mold. Additionally, we focused our studies on early time points (5 to 15 min) after adjuvant introduction, to best determine whether the curing rate was affected by adjuvant application. We did not study later time points like 30 min, since that is the time at which the company describes the cast material as being “fully cured.” Due to the lack of differences between the ultrasound gel- and liquid soap-treated samples as compared to control samples at early time points, we believe that the conclusions made in this study with regard to those agents would prevail at later time points as well. As mentioned previously, however, it could be of interest to investigate outcomes at longer curing times to determine whether alcohol sanitizer-treated samples would eventually develop strength profiles equivalent to control-treated samples.

## Conclusions

In summary, our work reveals that adjuvants like liquid hand soap and ultrasound gel provide no noticeable improvement in fiberglass cast material mechanics, while alcohol-based foam sanitizer significantly reduced cast material stiffness, yield stress, and ultimate load. These results indicate that clinical use of these adjuvants with the goal of improving mechanical properties is not supported and, in the case of alcohol-based foam sanitizers, could potentially increase the risk of cast failure. More work is needed to evaluate the clinical use of adjuvants in cast application and their long-term effects on cast mechanics to improve patient care and healthcare spending.

## Additional file

Additional file 1: **Table S1.** Survey Results. (DOCX 14 kb)

## Data Availability

All data and relevant material will be made available upon request.
