# Supplementary material for: Commonly used adjuvants (liquid soap, foam sanitizer, or ultrasound gel) do not improve strength or curing time of fiberglass cast material
Source: J Orthop Surg Res. 2019 May 30;14:166. doi: 10.1186/s13018-019-1202-1 (PMC6543659; doi:10.1186/s13018-019-1202-1)
Supplement: Supplementary file 1 — Table S1. Survey Results. (DOCX 14 kb) [file 13018_2019_1202_MOESM1_ESM.docx]

**Additional file 1: Table S1: Survey Results**

| **Question** | **Response (# / total responses)** |
| --- | --- |
| Do you apply fiberglass casts regularly in your practice? | Yes: 35/47  No: 12/47 |
| If you have applied a fiberglass cast, have you applied any of the following agents to the fiberglass cast material during application of the cast (select all that apply) | I don’t apply any additional agents: 14/35  I do apply additional agents: 21/35   - Liquid soap (hand soap): 9/21 - Foam hand sanitizer: 16/21 - Ultrasound jelly: 5/21 - Other: 4/21 |
| What is the reason for the application of additional agent (select all that apply) | Faster curing time: 8/21  Easier to mold cast: 4/21  Easier to laminate cast: 18/21  Other: 3/21 |
